# Pectin methylesterase 31 is transcriptionally repressed by ABI5 to negatively regulate ABA-mediated inhibition of seed germination

**DOI:** 10.3389/fpls.2024.1336689

**Published:** 2024-02-02

**Authors:** Yang Xiang, Chongyang Zhao, Qian Li, Yingxue Niu, Yitian Pan, Guangdong Li, Yuan Cheng, Aying Zhang

**Affiliations:** ^1^ College of Life Sciences, State Key Laboratory of Crop Genetics & Germplasm Enhancement and Utilization, Nanjing Agricultural University, Nanjing, China; ^2^ Sanya Institute of Nanjing Agricultural University, Nanjing Agricultural University, Sanya, China

**Keywords:** abscisic acid, seed germination, PME31, ABI5, transcription repression

## Abstract

Pectin methylesterase (PME), a family of enzymes that catalyze the demethylation of pectin, influences seed germination. Phytohormone abscisic acid (ABA) inhibits seed germination. However, little is known about the function of PMEs in response to ABA-mediated seed germination. In this study, we found the role of *PME31* in response to ABA-mediated inhibition of seed germination. The expression of *PME31* is prominent in the embryo and is repressed by ABA treatment. Phenotype analysis showed that disruption of *PME31* increases ABA-mediated inhibition of seed germination, whereas overexpression of *PME31* attenuates this effect. Further study found that ABI5, an ABA signaling bZIP transcription factor, is identified as an upstream regulator of PME31. Genetic analysis showed that PME31 functions downstream of ABI5 in ABA-mediated seed germination. Detailed studies showed that ABI5 directly binds to the *PME31* promoter and inhibits its expression. In the plants, *PME31* expression is reduced by ABI5 in ABA-mediated seed germination. Taken together, *PME31* is transcriptionally inhibited by ABI5 and negatively regulates ABA-mediated seed germination inhibition. These findings shed new light on the mechanisms of PMEs in response to ABA-mediated seed germination.

## Introduction

Seed germination is a vital step in plant growth and development and impacts the crop yield. The timing and rate of seed germination are controlled by a variety of internal and external factors (Graeber et al., 2012; Mei et al., 2023). Abscisic acid (ABA) and gibberellin acid (GA) are the primary internal factors that regulate the transition from dormancy to germination (Singh and Datta, 2023). ABA promotes seed dormancy and represses seed germination. GA is essential for the release of dormancy and for the initiation of germination. They antagonistically modulate seed germination, which is at least in part regulated by the balance and sensitivity to ABA and GA (Shu et al., 2013; Loades et al., 2023). In adverse environmental conditions, such as drought, high salinity, and extreme temperatures, ABA dominates the dormant state. The mechanisms of ABA-mediated seed germination inhibition have been investigated (Graeber et al., 2012; Tuan et al., 2018; Sharma et al., 2023). For instance, ABA reduces the water potential on the seed surface and water uptake to hinder seed germination (Berry and Bewley, 1992; Grainge et al., 2022). ABA also represses cell division and regulates the ion transport of seeds in seed germination (Liu et al., 2022). Besides, ABA modulates gene expression and regulates the activity of enzymes, such as amylases and protein kinases, to control seed germination (Xue et al., 2021; Li et al., 2022; Loades et al., 2023).

ABA initiates the cell response by activating downstream signaling genes. Several components have been identified in ABA signaling pathway, including PYRABACTIN RESISTANCE (PYR1)/PYR1-LIKE (PYL)/REGULATORY COMPONENT OF ABSCISIC ACID RECEPTOR (RCAR), SNF1-related protein kinase 2s (SnRK2s), protein phosphatases 2C (PP2Cs), ABSCISIC ACID INSENSITIVE3 (ABI3), ABSCISIC ACID INSENSITIVE4 (ABI4), and ABSCISIC ACID INSENSITIVE5 (ABI5) (Yan et al., 2020; Zhao et al., 2020). Among these, bZIP transcription factor ABI5 performs a crucial part in ABA-mediated seed germination (Lopez-Molina et al., 2001; Liu and Stone, 2010; Rushton et al., 2012; Nie et al., 2022). The *abi5* mutants showed insensitivity to ABA in seed germination, while *ABI5* overexpressors exhibited hypersensitivity (Lopez-Molina et al., 2001), suggesting that ABI5 positively regulated ABA-mediated seed germination inhibition. Studies showed that ABI5 directly regulated downstream target genes by binding to the G-box (ACGT) motif (Bi et al., 2017; Zhao et al., 2020). Bi et al. (2017) discovered that ABI5 was bound to the G-box of *CAT1* promoter and activated *CAT1* expression during seed germination. ABI5 also modulated the expression of ABA receptor genes (*PYL11* and *PYL12*) by directly binding to their promoters in ABA-mediated seed germination (Zhao et al., 2020). A key negative regulator of GA signaling DELLA protein RGL2 was directly bound to the *ABI5* promoter to promote its expression (Liu et al., 2016). Furthermore, ABI5 interacted with AFP, KEG, and MIEL1 in regulating seed germination (Liu and Stone, 2010; Nie et al., 2022). These studies demonstrated that ABI5 modulated seed germination in various manner.

Pectin methylesterase (PME) catalyzes the demethylation of pectin and alters cell wall structure and characteristics. The *PME* gene family is a large and diverse group with at least 66 members in *Arabidopsis thaliana* (Weber et al., 2013). Approximately 75% of Arabidopsis *PME* genes had tissue- and stress-specific expression profiles. The *PME* expression pattern showed the diversity of their involvement in cell wall modification throughout the development as well as stress responses (Pelloux et al., 2007; Huang et al., 2017). *PMEs* influence plant growth and development by changing the biomechanical features of the cell wall (Hongo et al., 2012; Zúñiga-Sánchez et al., 2014; Huang et al., 2017; Zhang et al., 2023). For instance, *PME35* reduced the degree of methylation in pectin and affected the mechanical characteristics of the stem cell wall (Hongo et al., 2012). Mutation of *PME3* influenced cell wall structure and zinc ion absorption (Weber et al., 2013). *PMEs* also play an important role in response to abiotic stresses. *PME34* improved plant heat tolerance by promoting stomatal opening, and increased water evaporation and temperature regulation capabilities under high-temperature conditions (Huang et al., 2017). *PME31* positively regulated salt stress tolerance (Yan et al., 2018). Brassinosteroids (BRs) modulated total PME activity in Arabidopsis by regulating *PME41* expression under chilling stress (Qu et al., 2011). Seed germination is intimately connected to the degree of pectin methylesterification. The demethylesterification of pectin promoted seed coat rupture and endosperm release (Müller et al., 2013). Zúñiga-Sánchez et al. (2014) found that the DUF642 gene *BIIDXI* improved PME activity to enhance Arabidopsis seed germination performance. In addition, PME activity was inhibited when garden cress seeds were treated with ABA (Scheler et al., 2015), indicating that ABA influenced PME activity in seed germination. However, the role of ABA and *PME* genes in seed germination is still unknown.

Here, we showed the role of PME31 in ABA-mediated seed germination inhibition. *PME31* negatively modulated ABA-mediated seed germination inhibition. We further identified the upstream regulator of *PME31*, an ABA-signaling bZIP transcription factor ABI5. We found that *PME31* expression was inhibited by ABI5 in ABA-mediated seed germination. Our findings shed light on the mechanisms of PMEs in response to ABA-mediated seed germination.

## Materials and methods

### Plant material and growth conditions

The Arabidopsis Biological Resource Center (ABRC) provided *Arabidopsis thaliana* L. Heynh. Ecotype Columbia (Col-0), Landsberg *erecta* (Ler-0), *pme31-1* (SALK_074820), *pme31-2* (CS25163), *abi3-1* (CS24), *abi4-1* (CS8104) and *abi5-1* (CS8105). The *abi5-1 pme31-1* double mutant was created through a genetic cross between *pme31-1* and *abi5-1*. The identified primers were listed in **Supplementary Table S1**. Seeds were planted in 1/2 MS medium and grew in a light incubator at 22°C/20°C (day/night), with a photoperiod of 16 h/8 h (day/night) and photosynthetic active radiation of 200 µmol m^–2^ s^–1^. Seeds were stratified at 4°C in darkness for various times (0, 12, 24, 36, 48 h) to detect *PME31* gene expression in seed germination. Samples were collected and promptly preserved in liquid nitrogen for analysis.

### Generation of transgenic plants

To generate *PME31*-overexpressing plants, the coding sequence of *PME31* with specific primers (**Supplementary Table S1**) was amplified and inserted into the pEarleyGate 101 vector from Earley et al. (2006). To investigate *PME31* tissue-specific expression, the *PME31* promoter sequence (2142 bp) was cloned into the pMD19-T vector (Takara, Japan) and inserted into the pGWB3 vector from Nakagawa et al. (2007) (**Supplementary Table S1**). The reporter *GUS* gene was driven by the *PME31* promoter (*proPME31:GUS*). The recombinant vectors were transformed into *Agrobacterium tumefaciens* strain GV3101. Transgenic plants were created by floral dip transformation (Clough and Bent, 1998). Positive plants were screened on 1/2 MS medium with 50 μg mL^-1^ Basta. Homozygous T3 transgenic plants were selected and collected for further analysis. *ABI5* overexpressors were obtained from the Arashare platform. *ABI5* was overexpressed by a β-estradiol-inducible promoter (Coego et al., 2014). *ABI4* overexpressor was obtained from Yan et al. (2020).

### Histochemical staining for β-glucuronidase (GUS) activity

The peeled seeds, rosette leaves, and roots of the *proPME31:GUS* transgenic plants were placed in GUS staining solution (10 mg mL X-Gluc, 0.1 M Na_2_HPO_4_-NaH_2_PO_4_ pH 7.0, 0.1% Triton X-100, 8 mM β-mercaptoethanol, 0.5 mM K_3_[Fe(CN)_6_], 0.5 mM K_4_[Fe(CN)_6_]) and vacuum-infiltrated for 30 min. The samples were stained at 37°C overnight and decolorized with 75% ethanol. The images were taken using a stereomicroscope (Optec SZ680, China).

### Real-time quantitative PCR (RT-qPCR) analyses

Total RNA was extracted from plant materials using TRIzol Reagent (CoWin Biotech, China). RNAs were then reverse-transcribed into cDNA by the 5×All-In-One MasterMix Kit (Abm Inc., China). The cDNA was amplified using the CFX96 Touch Real-Time PCR System (Bio-Rad, USA). The reaction system was 20 μL, containing 10μL AceQ qPCR SYBR Green Master Mix (Vazyme, China), 1.0 μL of each primer, 100 ng cDNA. The amplification conditions were as follows: 95 ° for 10 min; 95 ° for 15 s; 60 ° for 1 min, 40 Cycles. The internal reference was *Actin2*. The details of the specific primers were listed in **Supplementary Table S1**. The relative expression of each gene was calculated using the 2^-ΔΔCt^ method (Livak and Schmittgen, 2001).

### Determination of PME activity

PME activity was determined using a PME activity assay kit (Thermo Fisher Scientific, USA). Samples were extracted from seeds with PBS buffer (0.01 M, pH7.4). The protein samples were centrifuged at 12,000g for 20 min and collected supernatants. Protein concentration was measured by Bradford assays (Bradford, 1976). Samples were measured according to the manufacturer’s instructions. After the reaction, the absorbance value was measured at 450nm. The PME activity was calculated using the standard curve.

### Assays for seed germination and cotyledon greening

Assays for seed germination and cotyledon greening were performed as previously described (Yan et al., 2020). Each transgenic plant had more than 100 seeds collected simultaneously. After sterilization, seeds were sown on 1/2 MS medium either with or without 0.5 μM ABA. Once the radicle had emerged from the testa, the seed germination rate was recorded every 12 h. After 72 h, the cotyledon greening rate was measured. To investigate PME31 in response to ABA at the seedling stage, five-day-old seedlings were transferred to 1/2 MS medium with or without 10 µM ABA and then kept in an incubator under normal conditions for 14 d. Time to 50% germination and the percentage of maximum germination were recorded.

### Yeast-one-hybrid assays (Y1H)

Y1H assays were performed as previously described (Zhou et al., 2020; Xiang et al., 2021a). The pLacZi-proPME31 and pB42AD-ABI5 constructs were transformed into yeast strain EGY48. Transformants were screened on SD/–Trp–Ura plates and cultured in SD/–Trp–Ura/Gal/Raf/X-Gal (80 μg mL^−1^) plates for color development. The β-galactosidase activity was detected using a β-Galactosidase Assay Kit (Beyotime, Shanghai, China). The β-galactosidase activity was calculated as previously described (Xiang et al., 2021a). The pLacZi and pB42AD vectors were used as the negative control.

### Electrophoretic mobility shift assays (EMSA)

EMSA was performed as previously described (Xiang et al., 2021b). The coding sequence of *ABI5* was inserted into the pET-30a vector (Novagen, USA). His-ABI5 proteins were expressed in *Escherichia coli* Rosetta (DE3) for 6 h at 24°C following being induced with 0.5 mM isopropyl-1-thio-β-D-galactopyranoside (IPTG). The recombined proteins were purified using the MagnetHis™ protein purification system (Promega, USA) and eluted with elution buffer (100 mM HEPES, 500 mM imidazole, pH7.5). The probe contained the ttaCACGTag sequence (−945 to −954 bp) and was labeled using an EMSA Probe Biotin Labeling Kit (Beyotime, Shanghai, China). EMSAs were performed using a Chemiluminescent EMSA Kit (Beyotime, Shanghai, China). The unlabeled probe (Comp) and the mutated probe (mComp) were used as competitors (**Supplementary Table S1**).

### Luciferase assays

The *PME31* promoter was inserted into the p1381-LUC vector from Xiang et al. (2021a) (**Supplementary Table S1**). The coding sequence of *ABI5* was recombined into the pCAMBIA1300-221-3×flag vector (generated by insertion of a *35S* promoter and a Flag tag into the pCAMBIA1300 vector) from Xiang et al. (2021a) (**Supplementary Table S1**). The recombinant plasmids were transformed into *Agrobacterium tumefaciens* strain GV3101. The *proPME31:LUC*-*35S:REN* reporter was infiltrated into tobacco (*Nicotiana benthamiana*) leaves. After infiltration for 72 h, the LUC signals were imaged using a Tanon 5200 multi-chemiluminescent imaging system (Tanon, China). The tobacco leaves were collected and frozen in liquid nitrogen. The luciferase activities were detected using a Dual Luciferase Reporter Gene Assay Kit (Yeasen, Shanghai, China). The LUC/REN ratio was used to calculate the relative promoter activity.

### Accession numbers

The genes in this study have the following IDs: *PME31*, AT3G29090; *ABI5*, AT2G36270; *Actin2*, AT3G18780; *ABI3*, AT3G24650; *ABI4*, AT2G40220.

## Results

### The expression pattern of *PME31* during seed germination

**Figure 1:**
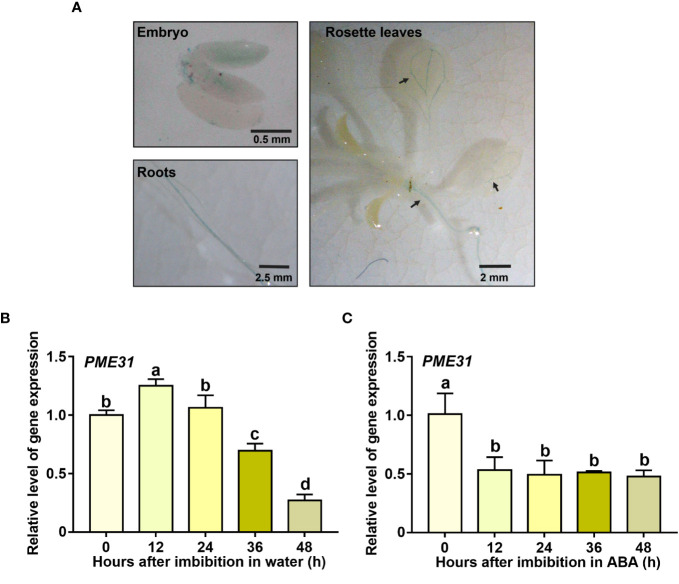
The expression pattern of *PME31* in different tissue and seed germination. **(A)** Tissue-specific expression analysis of *PME31* using GUS histochemical staining. The peeled seeds were obtained from Arabidopsis seed at 12 h imbibition. The rosette leaves and roots were obtained from 10-day-old Arabidopsis seedlings on 1/2 MS medium. The tissues were stained in GUS staining solution and vacuum-infiltrated for 30 min. The samples were stained at 37°C overnight and decolorized with 75% ethanol. The images were taken. **(B)** The expression of *PME31* during seed germination. Seeds were imbibed at 25°C in darkness for various times (0, 12, 24, 36, 48 d). **(C)** The expression of *PME31* in the presence of ABA during seed germination. Seeds were imbibed at 25°C in darkness and treated with 0.5 μM ABA. The gene expression of *PME31* was determined using RT-qPCR. *Actin2* was used as the internal reference. Data are means (± SD) of three biological replicates. Different letters indicate significant differences at P < 0.05 according to two-way ANOVA (Tukey’s multiple comparison test).

To investigate the tissue-specific expression of *PME31*, transgenic plants with *PME31* promoter-driven GUS reporters were generated. Different tissues from various development stages were collected and stained by GUS histochemical staining. The peeled seeds were obtained from Arabidopsis seed at 12 h imbibition. The Arabidopsis seed embryo exhibited a blue color. When Arabidopsis grew to 10 days old, the rosette leaves and roots were stained by GUS and showed that the leaf vasculature and primary root displayed a blue color (**Figure 1A**). The results suggested *PME31* was expressed in embryo, leaf vasculature, and primary root in Arabidopsis.

We next determined the expression pattern of *PME31* during seed germination. After imbibition, the *PME31* expression had a significant accumulation at 12 h imbibition and then decreased at 36 h and 48 h imbibition compared with dry seeds (**Figure 1B**). It suggested that *PME31* have an impact on seed germination. Previous studies showed that seed germination is tightly controlled by ABA (Zhao et al., 2020). To investigate whether *PME31* is involved in ABA-mediated seed germination, seeds were treated with ABA. The *PME31* expression decreased in ABA treatment for 12 h and was maintained until 48 h. The results showed that *PME31* expression was downregulated under ABA treatment (**Figure 1C**).

### Disruption of *PME31* promotes ABA-mediated inhibition of seed germination

**Figure 2:**
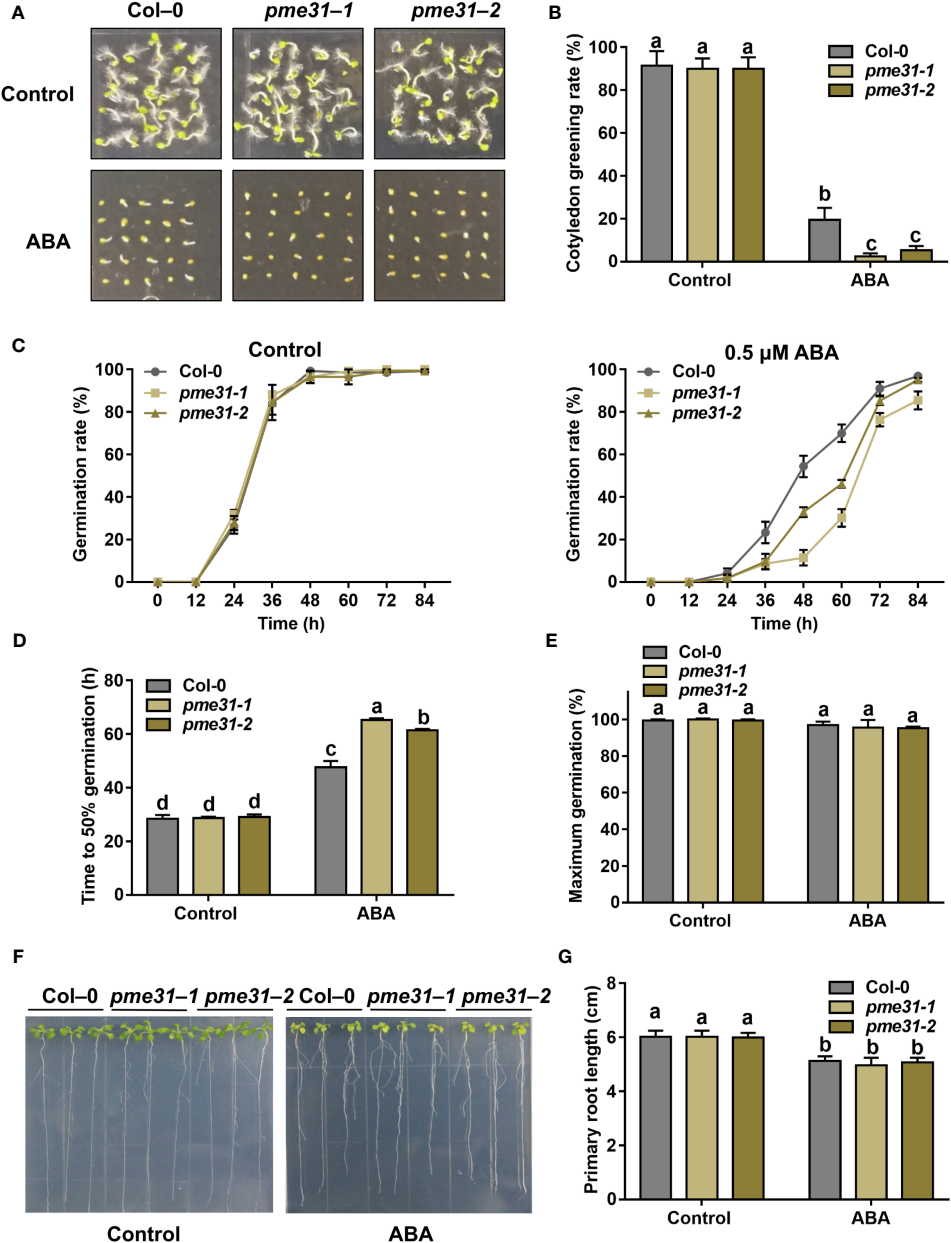
Disruption of *PME31* improves ABA-mediated inhibition of seed germination. **(A)** Phenotype of seed germination in Col-0 and *pme31* mutants treated with ABA. Seeds were sown on 1/2 MS medium with or without 0.5 µM ABA, and photographs were taken at 48 h of seed germination. **(B)** Green cotyledons rate was recorded after 72 h. **(C)** Germination rate of Col-0 and *pme31* mutants. Seeds were sown on 1/2 MS medium with or without 0.5 μM ABA. The seed germination rate was recorded every 12 h. **(D)** Time to 50% gemination of Col-0 and *pme31* mutants. **(E)** The percentage of maximum germination of Col-0 and *pme31* mutants. Seeds were sown on 1/2 MS medium with 0.5 μM ABA. **(F)** Phenotype of seedlings in Col-0 and *pme31* mutants treated with ABA. Five-day-old seedlings were selected and transferred to 1/2 MS medium with or without 10 µM ABA and placed in an incubator with normal conditions for 14 d. **(G)** Primary root length was recorded after growing for 14 d. Data are means (± SD) of three biological replicates. Different letters indicate significant differences at P < 0.05 according to two-way ANOVA (Tukey’s multiple comparison test).

To investigate whether *PME31* is involved in ABA-mediated seed germination, we screened two *pme31* mutants by PCR identification. The insertion site of the *pme31-1* mutant was in exon, while that of the *pme31-2* mutant was in 5’-UTR. RT-qPCR analysis showed that the expression of *PME31* decreased in the *pme31-2* mutant (**Supplementary Figure S1**). Thus, *PME31* was knocked out in the *pme31-1* mutant and knocked down in the *pme31-2* mutant. Under normal conditions, the *pme31-1* and *pme31-2* mutants had similar cotyledon greening rates and germination rates compared with the Col-0. However, the cotyledon greening rate and germination rate of *pme31-1* and *pme31-2* mutants were significantly lower than that of Col-0 under ABA treatment (**Figures 2A–C**). Moreover, the time to 50% germination of *pme31-1* and *pme31-2* mutants was higher compared with the Col-0 under ABA treatment (**Figure 2D**). The percentage of maximum germination had no significant difference in the Col-0, *pme31-1*, and *pme31-2* mutants (**Figure 2E**). The results suggested that disruption of *PME31* increased sensitivity to ABA during seed germination.

To further test whether *PME31* respond to ABA in the seedling stage, five-day-old seedlings were treated with ABA. Under normal conditions, the growth phenotype of *pme31-1* and *pme31-2* seedlings was not significantly different from Col-0. When supplemented with 10 µM ABA, there was also no significant difference in Col-0, *pme31-1*, and *pme31-2* seedlings (**Figures 2F, G**), indicating that the *PME31* mutation did not affect sensitivity to ABA in Arabidopsis seedlings. The above results suggested that disruption of *PME31* improves ABA-mediated inhibition of seed germination.

### Overexpression of *PME31* attenuates ABA-mediated inhibition of seed germination

**Figure 3:**
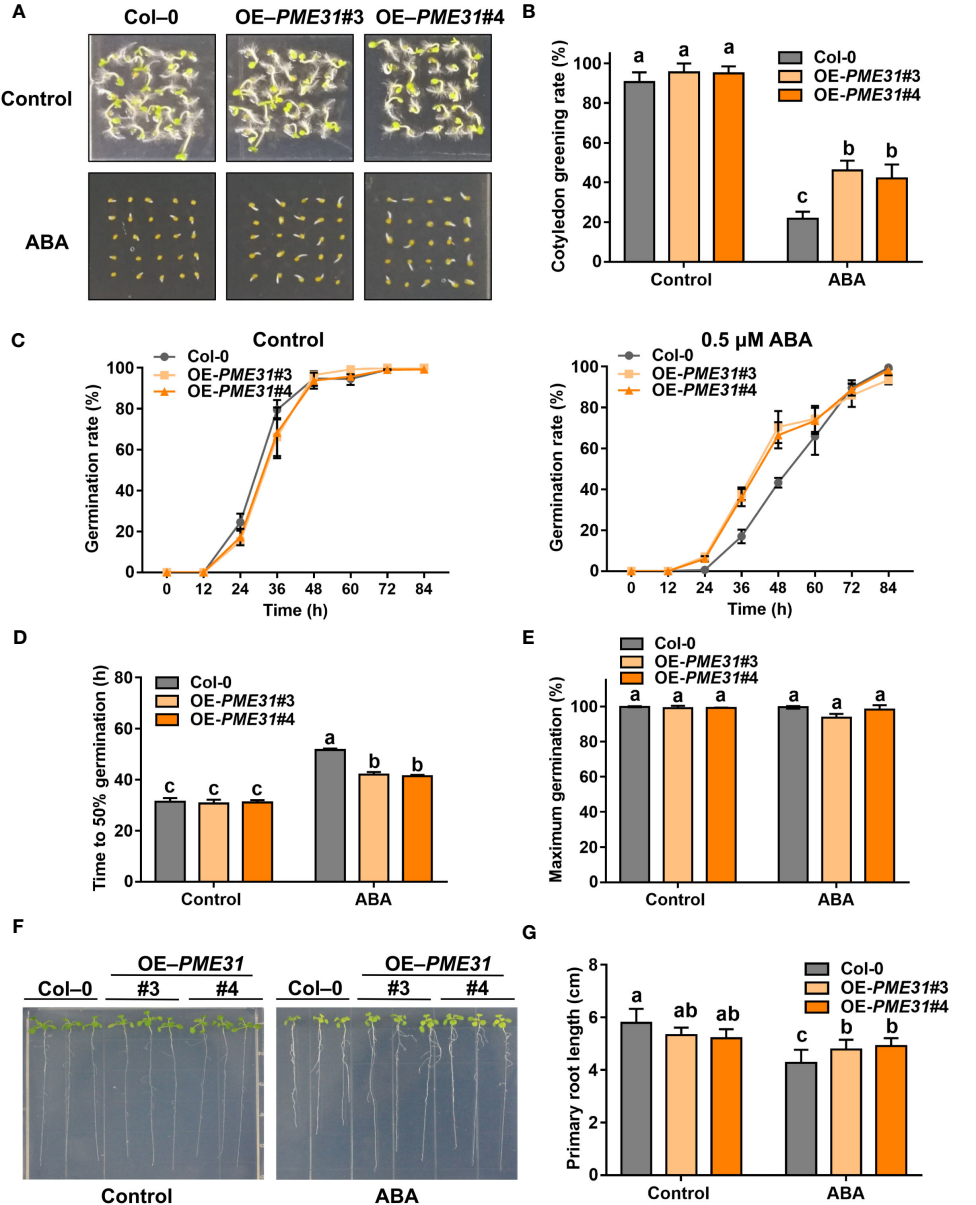
*PME31* overexpression reduces ABA-mediated inhibition of seed germination. **(A)** Phenotype of seed germination in Col-0 and *PME31* overexpressors treated with ABA. Seeds were sown on 1/2 MS medium with or without 0.5 µM ABA, and photographs were taken at 48 h of seed germination. **(B)** Green cotyledons rate was recorded after 72 h. **(C)** Germination rate of Col-0 and *PME31* overexpressors mutants. Seeds were sown on 1/2 MS medium with or without 0.5 μM ABA. The seed germination rate was recorded every 12 h. **(D)** Time to 50% gemination of Col-0 and *PME31* overexpressors. **(E)** The percentage of maximum germination of Col-0 and *PME31* overexpressors. Seeds were sown on 1/2 MS medium with 0.5 μM ABA. **(F)** Phenotype of seedlings in Col-0 and *PME31* overexpressors treated with ABA. Five-day-old seedlings were selected and transferred to 1/2 MS medium with or without 10 µM ABA and placed in an incubator with normal conditions for 14 d. **(G)** Primary root length was recorded after growing for 14 d. Data are means (± SD) of three biological replicates. Different letters indicate significant differences at P < 0.05 according to two-way ANOVA (Tukey’s multiple comparison test).

To further elucidate the role of *PME31* in ABA-mediated seed germination, we created transgenic plants overexpressing *PME31* under the control of the *35S* promoter (**Supplementary Figure S2A**). RT-qPCR analysis showed that transgenic plants overexpressing *PME31* (OE-*PME31*#3 and OE-*PME31*#4) had higher *PME31* expression (**Supplementary Figure S2B**). Under normal conditions, *PME31* overexpressors showed a similar cotyledon greening rate and germination rate as Col-0. Under ABA treatment conditions, the cotyledon greening rate and germination rate in *PME31* overexpressors were significantly higher than that in Col-0 (**Figures 3A–C**). The time to 50% germination of *PME31* overexpressors was lower compared with the Col-0 under ABA treatment (**Figure 2D**). The percentage of maximum germination had no significant difference in the Col-0 and *PME31* overexpressors (**Figure 2E**). It indicated that *PME31* overexpression reduced ABA-mediated inhibition of seed germination.

We then investigated the effect of *PME31* overexpression on Arabidopsis seedlings treated with ABA. Under normal conditions, the primary root length of OE-*PME31*#3 and OE-*PME31*#4 was similar to that of Col-0, but with the addition of 10 µM ABA, the primary root length of OE-*PME31*#3 and OE-*PME31*#4 was longer than that of the Col-0 (**Figures 3F, G**). Those results showed that *PME31* overexpression reduces ABA-mediated inhibition of seed germination.

### ABI5 directly binds to the *PME31* promoter and inhibits its expression

To identify upstream regulators of PME31, the *PME31* promoter was used as bait in a yeast-one-hybrid (Y1H) screen. The results showed that a bZIP transcription factor ABI5 was identified. ABI5 plays a vital role in seed germination, early seedling growth, and abiotic stresses in the presence of ABA (Skubacz et al., 2016). To investigate whether *PME31* was regulated by other ABI transcription factors, we analyzed the *PME31* expression in seeds of *abi3-1*, *abi4-1* mutants, and *ABI4* overexpressor and found that the *PME31* expression had no significant difference in those mutants (**Supplementary Figure S4**).

**Figure 4:**
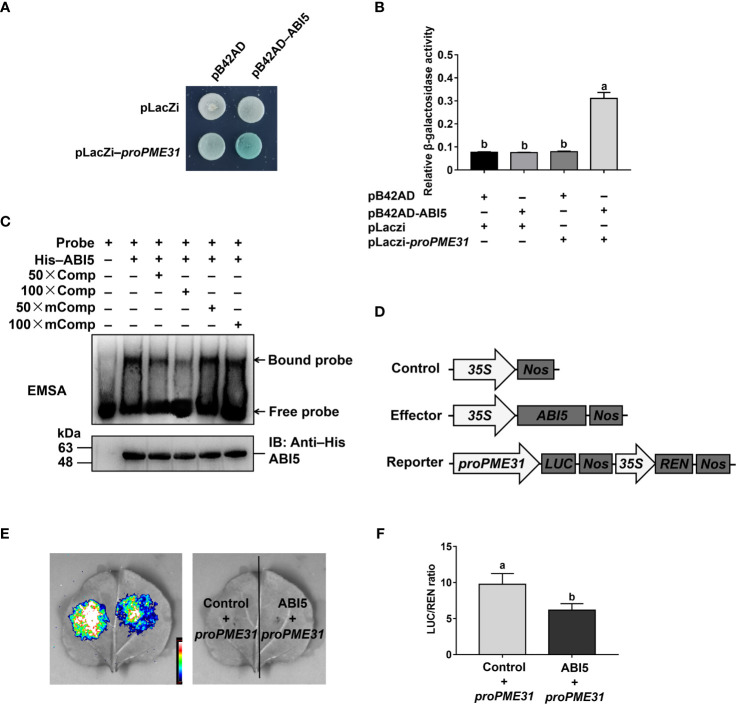
ABI5 directly binds to the *PME31* promoter and represses its expression. **(A)** Yeast-one-hybrid assays showing ABI5 directly binding to the promoter of *PME31.* The pLacZi-*proPME31* constructs were transformed with pB42AD-ABI5 into yeast strain EGY48. Transformants were screened on SD/–Trp–Ura plates and cultured on SD/–Trp–Ura/Gal/Raf/X-Gal (80 μg mL^−1^) for blue color development at 30°C for 3 d. The pLacZi and pB42AD vectors were used as the negative control. **(B)** β-Galactosidase activity in **(A)**. **(C)** Electrophoretic mobility shift assays showing ABI5 directly binding to the promoter of *PME31.* The 50-bp oligonucleotide containing the ttaCACGTag sequence (−945 to −954 bp) was synthesized and labeled as a probe. The 50× and 100× unlabeled probes (Comp) and 100× mutated probes (mComp) were used as competitors. **(D)** Schematic diagram of the effector and reporter constructs. The coding sequence of ABI5 was inserted into the pCAMBIA1300-221-3×flag vector to generate an effector. The *PME31* promoter was fused with LUC to generate a reporter. **(E)** Luciferase assays showing ABI5 acted as a transcriptional repressor of *PME31*. The *proPME31:LUC-35S:REN* reporter was infiltrated into tobacco leaves. The LUC signals were imaged. **(F)** LUC and REN activities. The relative activity of the promoter was calculated by the LUC/REN ratio. Data are means (± SD) of three biological replicates. Different letters indicate significant differences at P < 0.05 according to two-way ANOVA (Tukey’s multiple comparison test).

We then performed Y1H assays to test if ABI5 directly binds to the *PME31* promoter. As shown in **Figure 4A**, the color turned blue in EGY48 yeast cells transformed with pB42AD-ABI5 and pLacZi-proPME31 constructs, but not in yeast cells with other constructs. β-Galactosidase assays showed that yeast cells transformed with pB42AD-ABI5 and pLacZi-proPME31 had higher β-galactosidase activity compared with yeast cells transformed with other constructs (**Figure 4B**), suggesting that ABI5 directly binds to the *PME31* promoter.

We next search ABI5 binding elements in the promoters of *PME31* by using the Plant Promoter Analysis Navigator (PlantPAN; http://PlantPAN2.itps.ncku.edu.tw) (Chow et al., 2016) and found that the putative binding sequence TTACACGTAG located in −945 to −954 bp. We performed EMSAs to verify whether ABI5 directly binds to the TTACACGTAG sequence of the *PME31* promoter. The 50-bp oligonucleotide containing the TTACACGTAG sequence (−945 to −954 bp) was synthesized and labeled as a probe. When the His-ABI5 protein was incubated with the labeled probe, the protein-DNA complex with a slower migration speed was observed. When the His-ABI5 protein was incubated with the unlabeled probe, the protein-DNA binding was suppressed, but not with the mutated probe (**Figure 4C**). These results showed that ABI5 directly binds to the TTACACGTAG sequence of the *PME31* promoter.

To test if ABI5 was an activator or a repressor of *PME31*, we then performed luciferase assays. The effector was generated by the coding sequence of *ABI5* inserted into the pCAMBIA1300-221-3×flag vector. The reporter was generated by the *PME31* promoter fusing with LUC (**Figure 4D**). Tobacco leaves were infiltrated with the *proPME31*:LUC-35S:REN reporter and the *35S:ABI5* effector. The results showed that ABI5 inhibited LUC activity under the control of the *PME31* promoter (**Figures 4E, F**). Overall, those results indicated that ABI5 directly binds to the *PME31* promoter and acts as a transcriptional repressor of the *PME31* gene.

### PME31 acts genetically downstream of ABI5 in ABA-mediated seed germination

**Figure 5:**
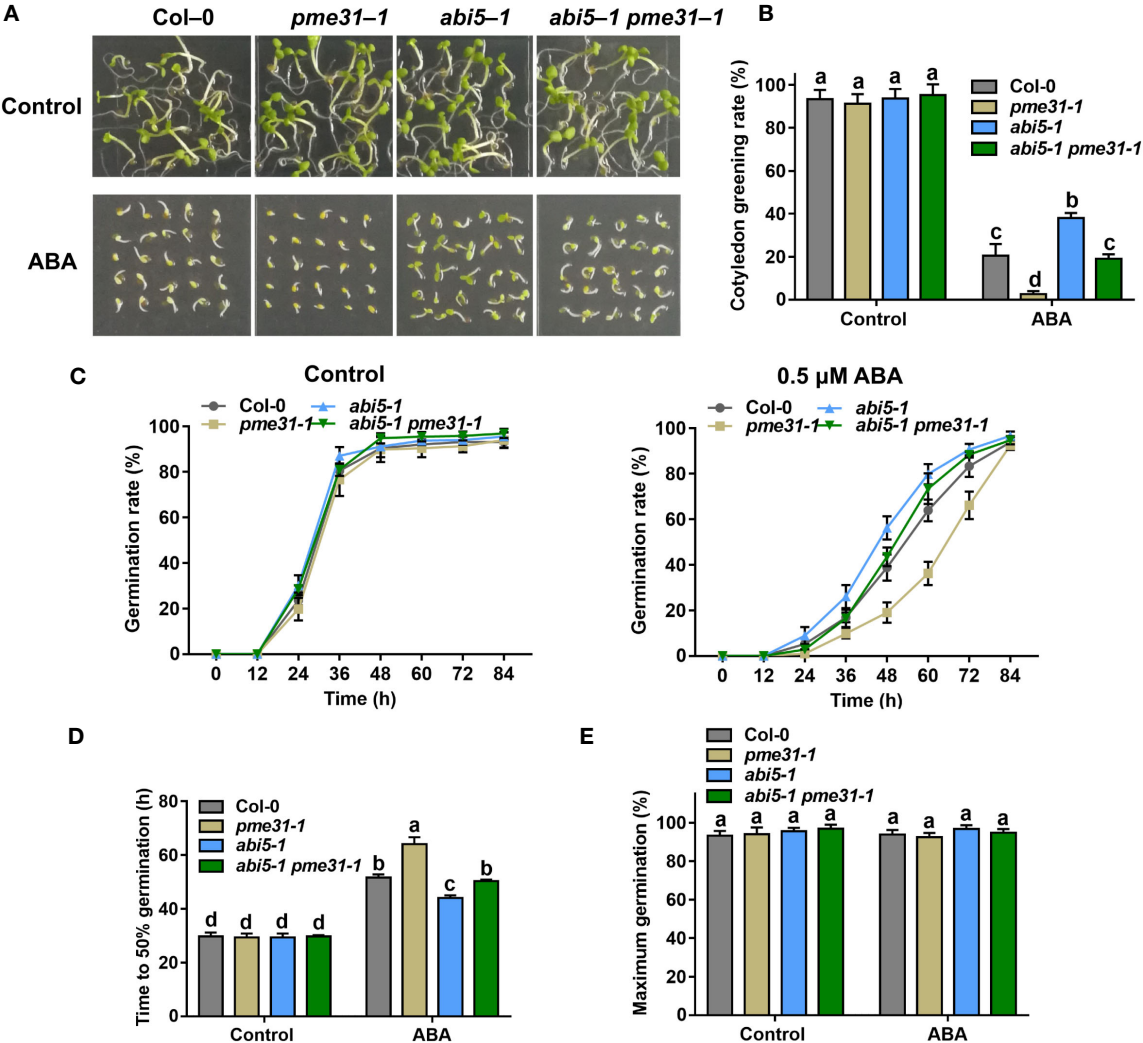
PME31 acts genetically downstream of ABI5 in ABA-mediated inhibition of seed germination. **(A)** Phenotype of seed germination in Col-0, *pme31-1*, *abi5-1, abi5-1 pme31-1* mutants treated with ABA. Seeds were sown on 1/2 MS medium with or without 0.5 µM ABA, and photographs were taken at 48 h of seed germination. **(B)** Green cotyledons rate was recorded after 72 h. **(C)** Germination rate of Col-0, *pme31-1*, *abi5-1, abi5-1 pme31-1* mutants. Seeds were sown on 1/2 MS medium with or without exogenous ABA (0.5 μM). The seed germination rate was recorded every 12 h. **(D)** Time to 50% gemination of Col-0, *pme31-1*, *abi5-1, abi5-1 pme31-1* mutants. **(E)** The percentage of maximum germination of Col-0, *pme31-1*, *abi5-1, abi5-1 pme31-1* mutants. Seeds were sown on 1/2 MS medium with 0.5 μM ABA. Data are means (± SD) of three biological replicates. Different letters indicate significant differences at P < 0.05 according to two-way ANOVA (Tukey’s multiple comparison test).

To dissect the genetic relationship between PME31 and ABI5, we crossed *pme31-1* with *abi5-1* and analyzed seed germination in Col-0, *pme31-1*, *abi5-1*, and *abi5-1 pme31-1* mutants under ABA treatment. Under normal conditions, the cotyledon greening rate and germination rate had no significant difference in *pme31-1*, *abi5-1*, and *abi5-1 pme31-1* compared with the Col-0. Under ABA treatment conditions, the cotyledon greening rate and germination rate of *abi5-1* were higher than that of the Col-0. It indicated *abi5-1* was insensitive to ABA-mediated inhibition of seed germination. The cotyledon greening rate and germination rate of *pme31-1* were lower than that of Col-0 and *abi5-1*, while that of *abi5-1 pme31-1* were intermediate between *pme31-1* and *abi5-1* (**Figures 5A–C**). The time to 50% germination of *abi5-1 pme31-1* mutant was more than that of *abi5-1* and less than that of *pme31-1* (**Figure 5D** *).* The percentage of maximum germination had no significant difference in those mutants (**Figure 5E**). Taken together, those results showed that PME31 acts genetically downstream of ABI5 in ABA-mediated seed germination.

### *PME31* expression is reduced by ABI5 in ABA-mediated seed germination

**Figure 6:**
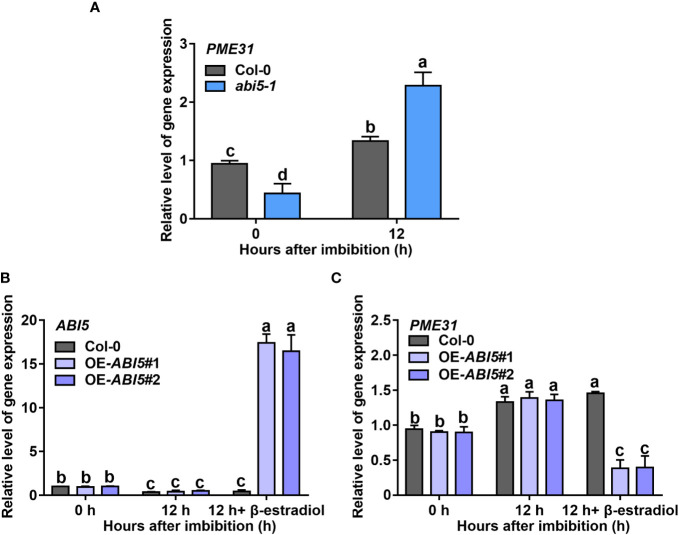
ABI5 decreases *PME31* expression in ABA-mediated seed germination **(A)** The expression of *PME31* in the *abi5-1* mutant. **(B)** The expression of *ABI5* in *ABI5* overexpressors. **(C)** The expression of *PME31* in *ABI5* overexpressors. Seeds were imbibed at 25°C in darkness for 12 h. The expression of *ABI5* was induced with or without 10 μM β-estradiol. *Actin2* was used as the internal reference. Data are means (± SD) of three biological replicates. Different letters indicate significant differences at P < 0.05 according to two-way ANOVA (Tukey’s multiple comparison test).

To further explore the effect of ABI5 on *PME31* expression in the plants, we examined the *PME31* expression in the *abi5-1* mutant and *ABI5* overexpressors during seed germination. Under normal conditions, *PME31* expression in the *abi5-1* mutant was significantly lower than in the wild-type. *PME31* expression increased significantly in the *abi5-1* mutant after 12 h imbibition. After 12 h imbibition, the *PME31* expression had a significant increase in the *abi5-1* mutant (**Figure 6A**). We then examined the *PME31* expression in *ABI5* overexpressors. *ABI5* overexpression was driven by a β-estradiol-inducible promoter. The *ABI5* expression was significantly increased in the presence of β-estradiol treatment (**Figure 6B**). After 12 h imbibition with β-estradiol induction, the *PME31* expression was significantly reduced in *ABI5* overexpressors compared with the Col-0 (**Figure 6C**). Therefore, ABI5 decreases *PME31* expression in seed germination.

## Discussion

Endospermic seeds, such as Arabidopsis, tobacco, and garden cress, often germinate in a two-step process: after the initial phase of water uptake by the dry seeds (imbibition), testa rupture (TR) occurs and is subsequently followed by endosperm rupture (ER) (Müller et al., 2006; Linkies et al., 2010). Cell walls are highly dynamic structures that provide mechanical support to plant cells. Hemicelluloses bind and crosslink with pectin in cell walls to generate a methylesterified hydrated matrix. Modifications of pectin are important to the process of TR and ER. PMEs regulate the methylesterification status of pectins by removing the methyl group of pectin. It results in an anionically charged matrix and alters the structure properties of the cell wall (Müller et al., 2013; Chandrasekaran et al., 2022). In garden cress, exogenous PME treatment increased testa permeability and promoted TR (Scheler et al., 2015). Salanenka et al. (2009) found that the activity of PMEs was associated with endosperm weakening in cucumber seeds, suggesting PMEs were closely related to seed germination. *PME* genes were more than 66 members in *Arabidopsis*. Most *PME* genes displayed tissue- and stress-specific expression profiles in plant development and stress responses (Pelloux et al., 2007; Weber et al., 2013; Huang et al., 2017; Tang et al., 2023). For instance, Weber et al. (2013) found that the root cell size in the *pme3* mutant seedlings was smaller in the presence of Zn^2+^. PbrPME44 greatly inhibited pollen tube growth and alleviated the increase in methyl-esterified pectin levels caused by PbrS–RNase (Tang et al., 2023). Previous studies showed that PME activity was close to seed germination. The increased PME activity improved the demethylesterification of pectin to facilitate the release of endosperm and promote seed germination (Müller et al., 2013). Scheler et al. (2015) found that ABA suppressed PME activity during seed germination. However, the mechanism of *PMEs* in response to ABA-mediated seed germination remains unknown. Studies showed that disruption of *PME31* reduced the release of seed coat mucilage and salt stress tolerance (Yan et al., 2018; Zhang et al., 2023), but the role of *PME31* in seed germination is not clear. In this study, we found that the *PME31* expression is prominent in the embryo and was downregulated in the presence of ABA (**Figure 1**). Phenotype analysis showed that *PME31* negatively regulated ABA-mediated seed germination inhibition (**Figures 2** and **3**). We further identified an upstream regulator of PME31, ABI5, which is an ABA signaling bZIP transcription factor (**Figure 4A**). *PME31* expression was downregulated in the *abi5-1* mutant, while it had a significant increase in the *abi5-1* mutant after 12 h imbibition. In seeds of *abi3-1*, *abi4-1* mutants, and *ABI4* overexpressor, the *PME31* expression had no significant difference (**Figure 6A**; **Supplementary Figure S4**). It suggested that *PME31* expression is mainly influenced by ABI5.

ABA inhibits seed germination and hinders post-germination growth at early developmental stages in plants. Studies have shown that ABI5 negatively regulated ABA-mediated seed germination (Lopez-Molina et al., 2001; Bi et al., 2017; Zhao et al., 2020). ABI5 interacted with a variety of factors, including functional proteins, transcription factors, and enzymes, to regulate seed germination. For instance, the circadian clock proteins PSEUDO-RESPONSE REGULATOR5 (PRR5) collaborated with ABI5 to increase ABA signaling and inhibit seed germination (Yang et al., 2021). In addition, ABA relieved the interaction of C-type Cyclin1;1 (CycC1;1) and inhibition of ABI5, which activated ABI5 activity in the ABA responses and inhibited seed germination (Guo et al., 2022). ABI5 also was bound to the target genes in ABA-mediated seed germination. ABI5 could bind to the promoters of *ABI3*, *CAT1*, and *PYR/PYL/RCAR* genes to influence their gene expression during seed germination (Lopez‐Molina et al., 2002; Bi et al., 2017; Zhao et al., 2020). However, whether ABI5 transcriptionally regulates *PMEs* expression is not clear. We found that ABI5 was directly bound to the *PME31* promoter and suppressed its expression (**Figure 4**). Meanwhile, genetic analysis revealed that ABI5 acted upstream of PME31 in ABA-mediated seed germination (**Figure 5**). Furthermore, ABI5 reduced *PME31* expression in plant seed germination (**Figure 6**). Consequently, it suggested that ABI5 was directly bound and downregulated *PME31* expression in ABA-mediated seed germination.

**Figure 7:**
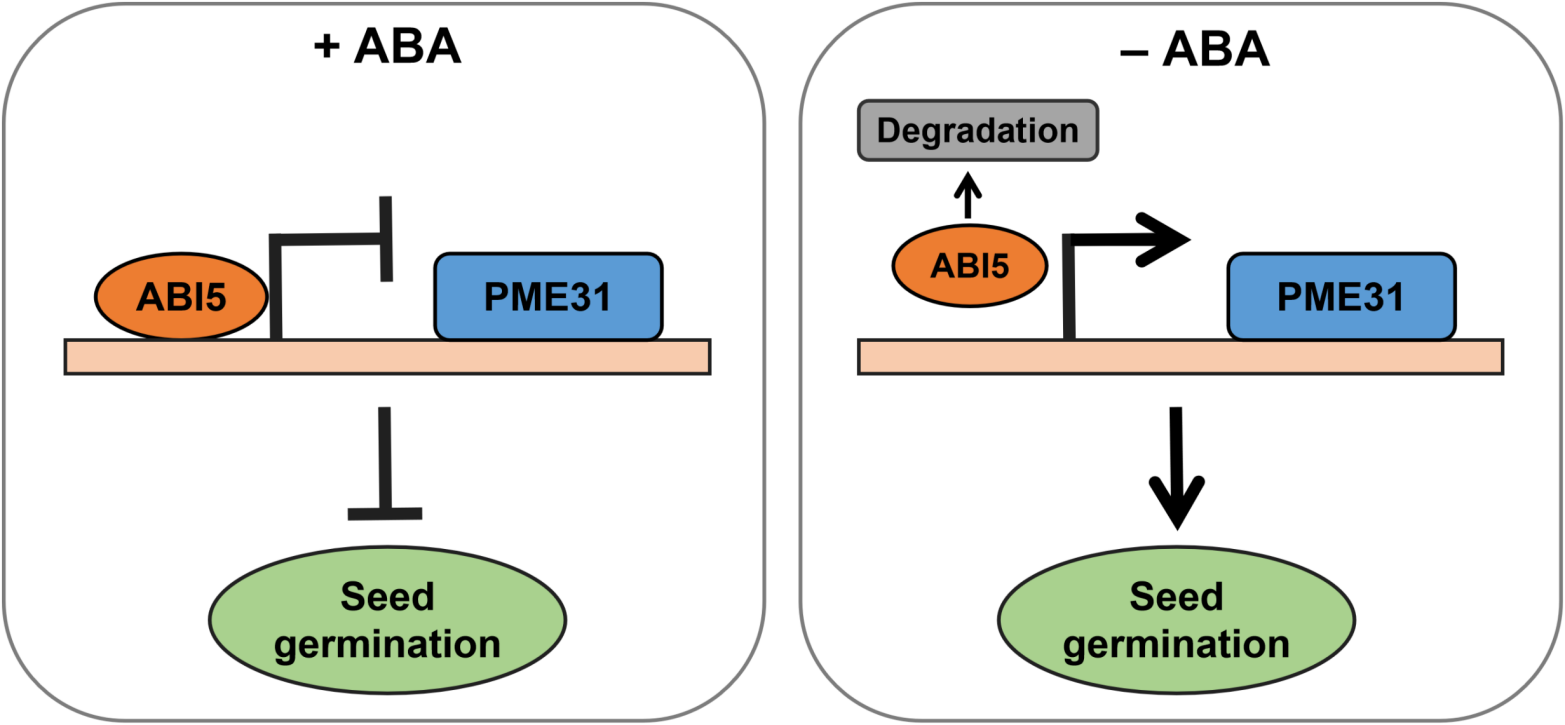
A working model for transcriptional repression of *PME31* by ABI5 in ABA-mediated seed germination As seed germination proceeded, ABA content and ABI5 protein level reduced, and the reduced ABI5 alleviated its transcriptional repression of *PME31*, thereby responding to the ABA-mediated seed germination.

The stability of ABI5 is strictly regulated as an important regulator of ABA-responsive genes during seed germination (Lopez-Molina et al., 2001; Nie et al., 2022). ABA elevated the stability of the ABI5 protein, but 26S proteasomes quickly broke down the protein when ABA was removed (Lopez-Molina et al., 2001). Numerous factors regulated the degradation of ABI5. For instance, ABI five binding protein (AFP) promoted ABI5 protein degradation (Lopez-Molina et al., 2003). ABA facilitated ABI5 accumulation by inducing the ubiquitination and proteasomal degradation of KEG (Liu and Stone, 2010). Moreover, the ubiquitin E3 ligase MIEL1 interacted with and ubiquitinated ABI5 to facilitate its degradation in the process of seed germination (Nie et al., 2022). It has been proved that as seeds germinate, the amount of ABA and the level of ABI5 protein gradually drop (Price et al., 2003; Zhao et al., 2020). We therefore hypothesized that as seed germination proceeds, ABA content and ABI5 protein level decrease, and the reduced ABI5 alleviates its transcriptional repression of *PME31*, thereby responding to the ABA-mediated seed germination (**Figure 7**).

In conclusion, *PME31* is transcriptionally repressed by ABI5 to negatively regulate ABA-mediated seed germination inhibition. Our findings shed light on the mechanisms that *PME31* is in response to ABA-mediated seed germination.

## Data availability statement

The original contributions presented in the study are included in the article/**Supplementary Files**, further inquiries can be directed to the corresponding author/s.

## Author contributions

YX: Data curation, Formal analysis, Funding acquisition, Investigation, Methodology, Software, Writing – original draft, Writing – review & editing. CZ: Data curation, Investigation, Writing – original draft, Writing – review & editing. QL: Data curation, Investigation, Writing – original draft, Writing – review & editing. YN: Data curation, Methodology, Writing – original draft, Writing – review & editing. YP: Data curation, Investigation, Methodology, Writing – original draft. GL: Data curation, Investigation, Methodology, Writing – original draft. YC: Data curation, Investigation, Methodology, Writing – original draft. AZ: Funding acquisition, Writing – review & editing.
