# Supplementary material for: Pectin methylesterase 31 is transcriptionally repressed by ABI5 to negatively regulate ABA-mediated inhibition of seed germination
Source: Front Plant Sci. 2024 Feb 2;15:1336689. doi: 10.3389/fpls.2024.1336689 (PMC10869471; doi:10.3389/fpls.2024.1336689)
Supplement: Supplementary file 1 [file DataSheet_1.docx]

Supplementary Material


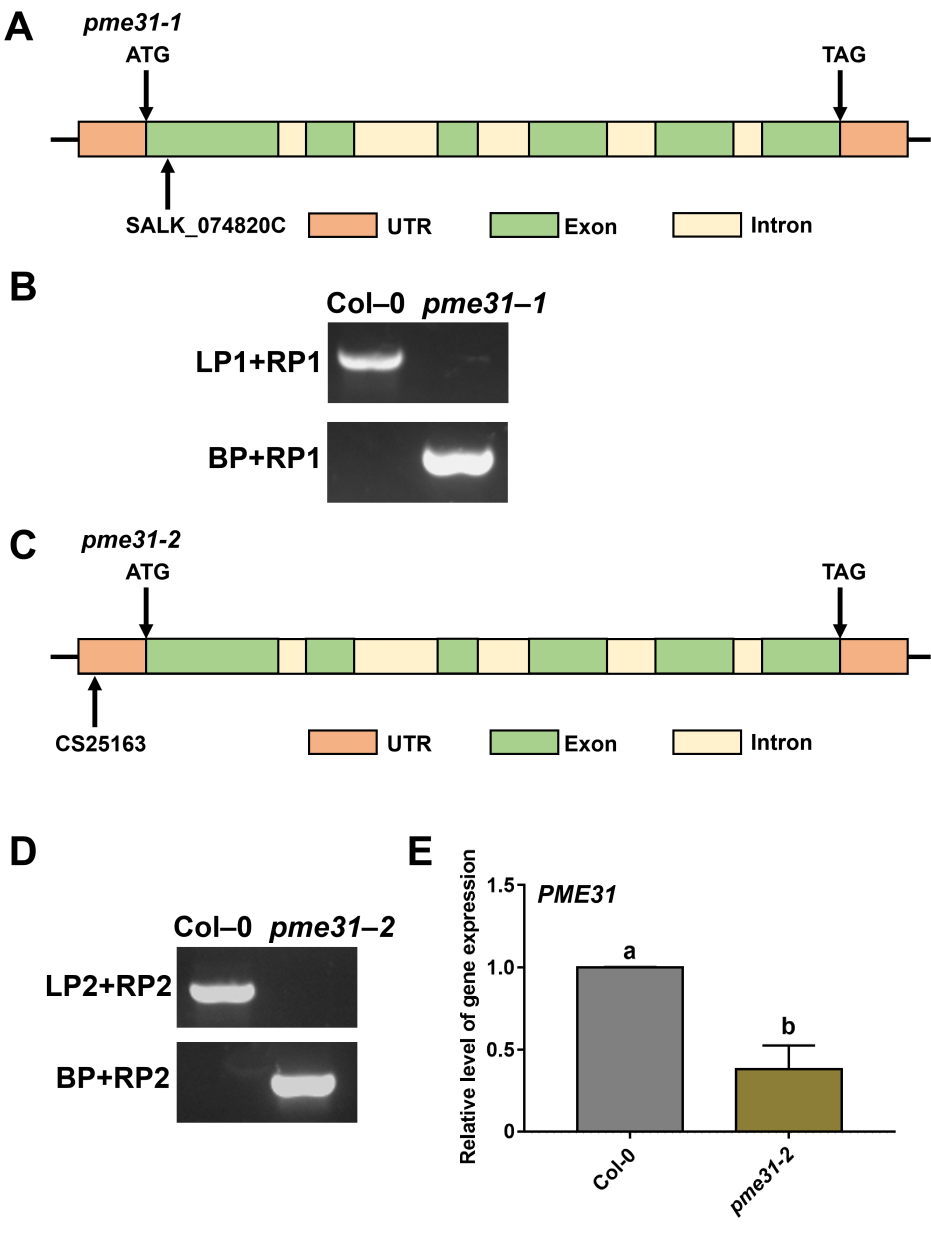


**Supplementary Figure S1** Acquisition and identification of *pme31* mutants. **(A)** Schematic diagram of the *PME31* insertion site in *pme31-1* mutant. **(B)** PCR identification of *pme31-1* mutant. **(C)** Schematic diagram of the *PME31* insertion site in *pme31-2* mutant. **(D)** PCR identification of *pme31-2* mutant. **(E)** The expression of *PME31* in *pme31-2* mutant was determined using RT-qPCR. *Actin2* was used as the internal reference. Data are means (±SD) of three biological replicates. Different letters indicate signiﬁcant differences at P < 0.05 according to two-way ANOVA (Tukey’s multiple comparison test).


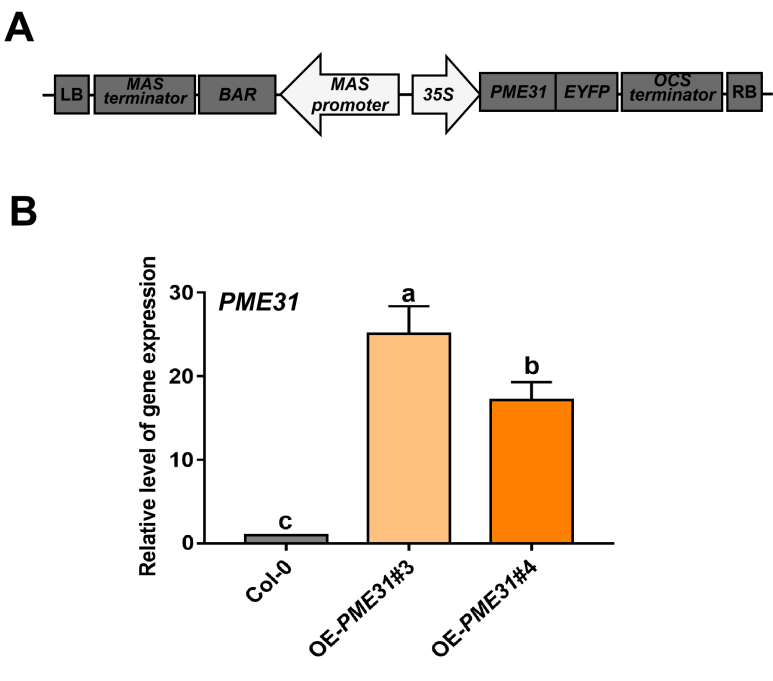


**Supplementary Figure S2** The expression of *PME31* in *PME31* overexpressors. **(A)** T-DNA region of the plasmid of *35S*:*PME31-MAS*:*BAR*. **(B)** The expression of *PME31* in *PME31* overexpressors was determined using RT-qPCR. *Actin2* was used as the internal reference. Data are means (±SD) of three biological replicates. Different letters indicate signiﬁcant differences at P < 0.05 according to two-way ANOVA (Tukey’s multiple comparison test).

**
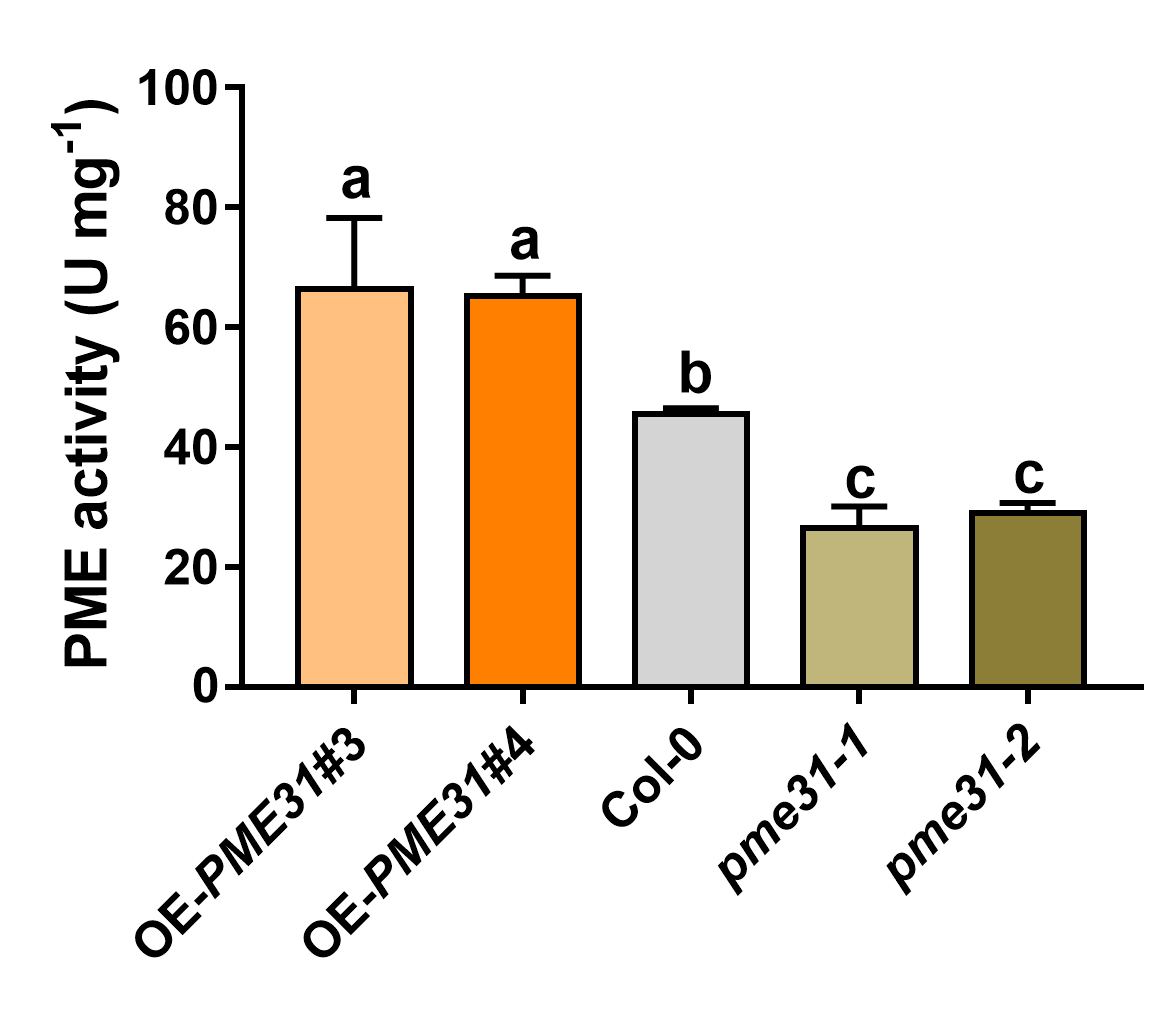
**

**Supplementary Figure S3** PME activity in OE-*PME31* and *pme31* mutants. Samples were extracted from seeds of OE-*PME31* and *pme31* mutants. PME activity was determinated by PMEs activity assay kits. Protein concentration was measured by Bradford assays. Data are means (±SD) of three biological replicates. Different letters indicate signiﬁcant differences at P < 0.05 according to two-way ANOVA (Tukey’s multiple comparison test).


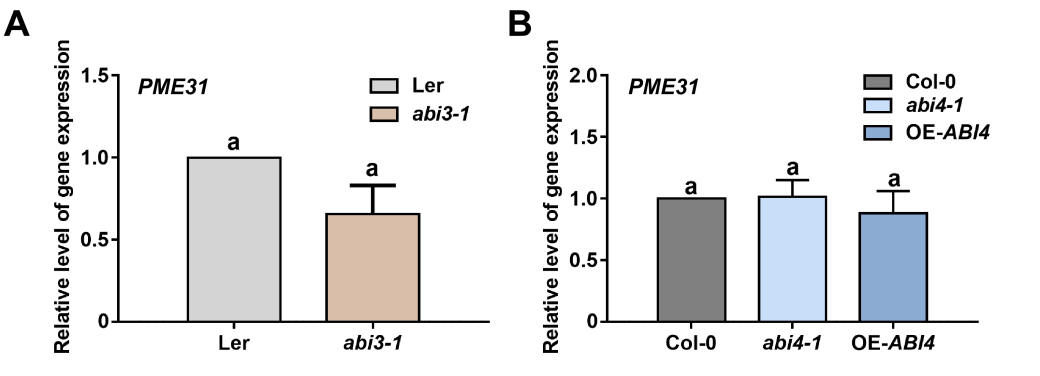


**Supplementary Figure S4 (A)** The expression of *PME31* in the seeds of wild-type, *abi3-1*, *abi4-1*, OE-*ABI4* mutants. The expression of *PME31* in *abi3-1* mutant*.* **(B)** The expression of *PME31* in *abi4-1* and OE-*ABI4* mutants. The expression of *PME31* was determined using RT-qPCR. *Actin2* was used as the internal reference. Data are means (±SD) of three biological replicates. Different letters indicate signiﬁcant differences at P < 0.05 according to two-way ANOVA (Tukey’s multiple comparison test).

**Table S1 Sequence information for primers used for this study.**

| **Primer Name** | **Vector** | **Sequence (5’-3’)** |
| --- | --- | --- |
| **Primers used in vector construction** | | |
| PME31-attB1 | pEarleygate 101 | ACAAGTTTGTACAAAAAAGCAGGCTTCACCATGATAATCGAAGTGAAATGC |
| PME31-attB2 | pEarleygate 101 | ACCACTTTGTACAAGAAAGCTGGGTCGAAATTCTTTATGGAAGATGAACT |
| proPME31-F | pMD19-T | TCAAGTATATATCTTAACTAACATTA |
| proPME31-F | pMD19-T | AGTTGTAAAACCCTAGTGAG |
| proPME31-GUS-F | pGWB3 | acaagtttgtacaaaaaagcaggcttcaccTCAAGTATATATCTTAACTAACATTA |
| proPME31-GUS-R | pGWB3 | accactttgtacaagaaagctgggtcAGTTGTAAAACCCTAGTGAG |
| **Primers for RT-qPCR** | | |
| PME31-RT-F |  | TCACCAGGCGTCTAGAGTCA |
| PME31-RT-R |  | GACTCGTATCGCCACAGCTT |
| ABI5-RT-F |  | TGGGTGACCCATCAGGTTATGC |
| ABI5-RT-R |  | TCTGAAGACACCGGGCTTAAC |
| Actin2-RT-F |  | ACTCTTTTTGTGTGTTTGCAGC |
| Actin2-RT-R |  | CAGCACAATACCGGTTGTACG |
| **Primers used in identification** | | |
| pme31-1-LP1 |  | TCAAATTTCACCTAGGTGATTTG |
| pme31-1-RP1 |  | CACAACCAAACGTACCAGTCC |
| pme31-2-LP2 |  | ATTATAAAAGGCGCAACGGTC |
| pme31-2-RP2 |  | TGCATGGTTTGAAATATGTGC |
| abi5-1-LP |  | GGTTATGCTAAAAGGACAGGAG |
| abi5-1-RP |  | GGACCATCCACTACTCTTTTC |
| BP |  | ATTTTGCCGATTTCGGAAC |
| OE-PME31-F |  | ATCGAGACAAGCACGGTCAA |
| OE-PME31-R |  | AAACCCACGTCATGCCAGTT |
| **Primers for Y1H assays** | | |
| pB42AD-ABI5-F | pB42AD | gattatgcctctcccgaattcATGGTAACTAGAGAAACGAAGTTGAC |
| pB42AD-ABI5-R | pB42AD | agaagtccaaagcttctcgagTTAGAGTGGACAACTCGGGTTC |
| pLacZi-proPME31-F | pLacZi | TTTGATATTGGATCGgaattcTCAAGTATATATCTTAACTAACATTA |
| pLacZi-proPME31-R | pLacZi | gtcgacagatccccgggtaccAGTTGTAAAACCCTAGTGAG |
| **Primers for EMSA** | | |
| proPME31-probe-F |  | TTGAACAAAGAACTGTTACACGTAGAACCAAACAATATGATGAAAAGTAT |
| proPME31-probe-R |  | ATACTTTTCATCATATTGTTTGGTTCTACGTGTAACAGTTCTTTGTTCAA |
| proPME31-mutant-F |  | TTGAACAAAGAACTGTTAaAaaaAGAACCAAACAATATGATGAAAAGTAT |
| proPME31-mutant-R |  | ATACTTTTCATCATATTGTTTGGTTCTtttTtTAACAGTTCTTTGTTCAA |
| His-ABI5-F | pET-30a | gccatggctgatatcggatccATGGTAACTAGAGAAACGAAGTTGAC |
| His-ABI5-R | pET-30a | ttgtcgacggagctcgaattcTTAGAGTGGACAACTCGGGTTC |
| **Primers for Luciferase assays** | | |
| flag-ABI5-F | pCAMBIA1300-221-3×flag | GACGATGATAAGGGCGGTACCATGGTAACTAGAGAAACGAAGTTGAC |
| flag-ABI5-R | pCAMBIA1300-221-3×flag | GTCCTAGGCTACGTAGGATCCTTAGAGTGGACAACTCGGGTTC |
| proPME31-F | p1381-LUC | ATTACGaattcccggggatccTCAAGTATATATCTTAACTAACATTA |
| proPME31-R | p1381-LUC | attgttgtaaaaataaagcttAGTTGTAAAACCCTAGTGAG |
